# New Anti SARS-Cov-2 Targets for Quinoline Derivatives Chloroquine and Hydroxychloroquine †

**DOI:** 10.3390/ijms21165856

**Published:** 2020-08-14

**Authors:** Davide Gentile, Virginia Fuochi, Antonio Rescifina, Pio Maria Furneri

**Affiliations:** 1 Dipartimento di Scienze del Farmaco, University of Catania, 95125 Catania, Italy; davide.gentile@unict.it; 2 Dipartimento di Scienze Biomediche e Biotecnologiche, University of Catania, 95125 Catania, Italy; vfuochi@unict.it

**Keywords:** SARS-CoV-2, chloroquine, hydroxychloroquine

## Abstract

The rapid spread of severe acute respiratory syndrome coronavirus 2 (SARS-CoV-2) has created a severe global health crisis. In this paper, we used docking and simulation methods to identify potential targets and the mechanism of action of chloroquine (CQ) and hydroxychloroquine (HCQ) against SARS-CoV-2. Our results showed that both CQ and HCQ influenced the functionality of the envelope (E) protein, necessary in the maturation processes of the virus, due to interactions that modify the flexibility of the protein structure. Furthermore, CQ and HCQ also influenced the proofreading and capping of viral RNA in SARS-CoV-2, performed by nsp10/nsp14 and nsp10/nsp16. In particular, HCQ demonstrated a better energy binding with the examined targets compared to CQ, probably due to the hydrogen bonding of the hydroxyl group of HCQ with polar amino acid residues.

## 1. Introduction

In recent years, two *β*-coronaviruses (CoVs) have caused outbreaks of pneumonia, the severe acute respiratory syndrome (SARS), and the Middle Eastern respiratory syndrome (MERS) [1]. COVID-19 is a disease caused by a new coronavirus known as severe acute respiratory syndrome coronavirus 2 (SARS-CoV-2), which leads to respiratory failures such as the previous SARS-CoV and MERS-CoV [2,3]. On 11 March, 2020, the World Health Organization characterized COVID-19 as a pandemic [4]. The outbreak started in mid-December 2019 in Wuhan, China [2,5]. Today, it is not possible to define with certainty the route taken by the virus to reach humans; but numerous assumptions agree with the animal origin [6,7,8]. Most likely, SARS-CoV-2 might have cryptically circulated within humans for years before being discovered [9].

Subsequently, due to the high contagiousness, this coronavirus spread worldwide very quickly [10]. CoVs belong to the subfamily Orthocoronavirinae of the Coronaviridae family, whose genome is a single-stranded, positive-sense RNA.

The reproductive cycle of coronaviruses is particularly elaborate and complex [11], and can be divided into two different translational and transcriptional moments, which are strongly linked to the action capacity of many antiviral drugs. Although numerous papers have been published on the activity and molecular mechanisms of different antiviral molecules, no molecules having a marked and specific activity have yet been found. Among the most discussed drugs of the last five months, CQ and its HCQ derivative have been the subject of numerous in vitro and clinical studies [12,13,14,15,16].

CQ was synthesized for the first time by H. Andersag in Bayer laboratories in Elberfeld in 1934 [17]. Initially studied also in vivo for its antimalarial activity, was abandoned in favor of quinacrine hydrochloride, a less toxic molecule [17,18]. Rediscovered in America during the Second World War, it was marketed in 1947 [17,19,20,21,22,23]. Its structural formula derives from a molecule of natural origin, quinine, from which it differs for the substituents at position 6 and the position linked to chain 4 [17,18]. Although CQ was used for many years as both antimalarial prophylaxis and therapy, its use has been significantly reduced due to the onset of resistance, especially in Plasmodium falciparum [24]. Its antimetabolic activity has been reported since the first studies with particular reference to the inhibition of numerous enzymatic pathways in plasmodia [24], and inhibition of DNA polymerase and RNA polymerase in bacteria [25,26,27], as well as in Tetrahymena pyriformis, a ciliated protozoa [28]. Although CQ targets plasmodia, unfortunately, the same inhibitory effects have been demonstrated in a model developed in vivo on rats [29]. However, the antiprotozoal mechanism was subsequently associated with its lysosomotropic characteristics and its interaction with hemozoin [30]. The antiviral activity, described in the early 1960s, was initially deduced from a clinical study on the therapy of viral hepatitis [31], and then described in *Mouse Hepatitis Virus* (MHV, *murine Beta Coronavirus*) experimentally [32]. In this case, the six-hour treatment with CQ of mouse peritoneal macrophages infected with MHV reduced the viral load compared to those of the untreated cells. However, after 72 h from the treatment, an increase in the viral load was noted.

Consequently, the author concluded that this phenomenon was due in some way to a higher permeability of lysosomes. The antiviral activity was later demonstrated in DNA phages [33]. CQ was also reported to inhibit other viruses such as *Encephalomyocarditis virus* (Cardiovirus), *Sindbis virus*, *Influenza A2*, *Newcastle disease virus*, *Herpes simplex*, and *Vaccinia* [34]. A study on the antiviral activity against myxoviruses allowed the authors to argue that probably the mechanism of action was to inhibit stripping through the so-called stabilization of lysosomal membranes [35]. In 1974, the first in vivo study on CQ activity was conducted in a model of infection with *Moloney Murine Sarcoma virus* (a murine retrovirus), which has shown that CQ inhibited the development of the tumor when preventively inoculated at a concentration of 50 mg/kg in the newborn mouse [36]. Numerous other reports were published regarding other animal and human viruses that attributed its antiviral activity to lysosomotropy [37,38,39,40]. However, this feature and its real antiviral activity has not been more confirmed in MHV [41]. Furthermore, some viruses, such as poliovirus, seem to be insensitive up to the concentration of 150 μM [42,43].

Particular interest has aroused the ability of CQ to inhibit both HIV replication and glycosylation of viral particles and, at the same time, to act in synergy with the viral protease [44]. Furthermore, impairment of ACE2 terminal glycosylation has been reported. In fact, at 25 μM CQ, although ACE2 is expressed in similar quantities at the cell surface, the variations in its glycosylation status might render the ACE2/SARS-CoV interaction less efficient and inhibit virus entry when the cells are treated with the drug [45]. The same authors have also shown inhibition of endosome-mediated viral entry.

Another quinoline derivative has been considered capable of inhibiting viral replication: In fact, the hydroxychloroquine, a well-known antimalarial drug recognized as a life-saving drug from the WHO (https://www.who.int/medicines/publications/essentialmedicines/en/), has also been used in the treatment of arthritic complications during some viral infections [46,47]. HCQ has also been shown to inhibit the replication of HIV by a mechanism similar to that of chloroquine [48,49]. A hypothetical anti-HPV activity of HCQ has also been proposed [50].

HCQ has recently been the subject of renewed interest both as a therapy for the disease caused by SARS-CoV-2 [51,52,53], and prophylaxis [54]. Interestingly, HCQ can intervene as a competitor for absorption by binding to the cellular receptor [55], or onto iron homeostasis [56].

Looking for possible antiviral molecules, many new targets have been found in numerous nonstructural proteins [57,58,59,60,61], but insufficient attention has been paid to the role of the nsp10/nsp14 complex [62], and the envelope (E) protein [63].

This study aimed to investigate the molecular interaction of CQ or HCQ with three nonstructural proteins of SARS-CoV-2 that could represent a new and more useful target of the two drugs. In fact, it has been recently suggested that nsp10 and nsp16 could be an innovative target for antiviral drug discovery [64]. These features could be an innovative and exciting explanation by which the mechanism of quinoline derivatives might be directed more to the late replicative process rather than to uncoating.

## 2. Results and Discussion

### 2.1. E Protein

E protein is a short peptide of 75 amino acids in SARS-CoV-2 [63]. It is organized in three different domains: *N*-terminal, transmembrane (with most hydrophobic amino acids), and *C*-terminal [63,65]. E protein is one of the major structural proteins of the coronavirus [66], and is essential for infection as well as for bending membranes, and playing a role in virus maturation processes [67]; in fact, in CoV assembly, the formation of the envelope requires the only expression of M and E and not N. Moreover, the ability of E and M to drive the formation of a virus-like particle clearly shows that E is essential for assembly [68]. Moreover, E protein interacts with the host proteins [69], as well as influences host cell membrane permeability [63,70], and it has been associated with different membrane topologies [63,68,71,72]. In fact, it has been described, with a low frequency, a version with an *N*-glycosylation [71] of asparagine in position 66 [72]. Its functions as an ion-channeling viroporin [73], shared with two other proteins [68,74,75], have been associated with inflammation observed in acute respiratory distress [76] as well as to endoplasmic reticulum stress. At the same time, its role in cellular apoptosis remains questionable [63]. In addition, the Ca^2+^ passage to E protein channel has been associated with the activation of NLSP3 inflammasomes [76,77]. Although not crucial for virus multiplication, the absence of E protein showed an accumulation of probably aberrant virions [78]. Its interaction with PALS1, a particular class of tight junction-associated protein, has been associated with damage to lung epithelium [79]. E protein may represent a novel strategy used by SARS-CoV to increase its virulence [80], and, although it is not involved in the innate immune response to SARS-CoV, it might have an important role in developing a vaccine strategy as well as might be an important target for a chemotherapeutic approach [66,80,81].

**Figure 1:**
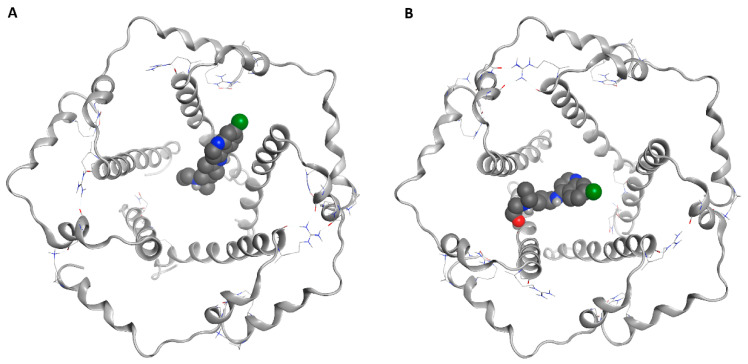
Docked poses of chloroquine (CQ) (**A**) and hydroxychloroquine (HCQ) (**B**) in the central cavity of the envelope (E) protein.

**Figure 2:**
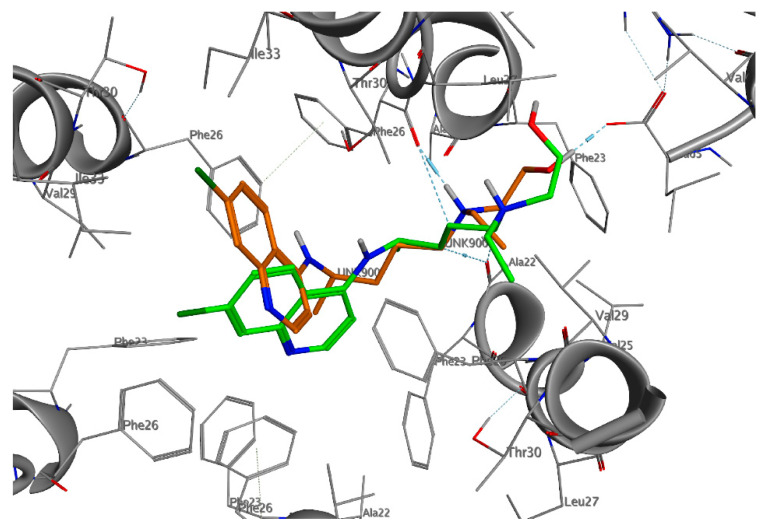
Three-dimensional (3D)-interaction diagram of HCQ in the docked pose (green) and average pose during molecular dynamics (MD) simulation (orange) with the E protein.

The great difficulty of crystallization for a hydrophobic protein did not allow the study and design of selective inhibitors for E protein. Given the low reliability of the homology model obtained and of the models present on the web, in addition to the lack of data in the literature on the design of inhibitors, we decided to investigate the entire pentameric structure using the NMR structure of E protein from SARS-CoV-1 [82]. A comparative sequence analysis via Multalin reveals that SARS-CoV-1 and SARS-CoV-2 E protein sequences share 94.7% identity amongst themselves. The results of the molecular docking (Figure 1, Figure 2, Figures S1 and S2) showed that HCQ has better binding energy (−8.6 kcal/mol) than CQ (−8.3 kcal/mol). The docking pose highlights that the interactions between HCQ and the E Protein are due to the hydrogen bond with the Phe23 residue, while the drug quinolinic region is stabilized by the hydrophobic bonds with the five-phenyl residues Phe26 in the central part of the ion channel (Figure 2 and Figure S2). Other hydrophobic interactions concern the aliphatic area of the drug with the residues Leu19, Ala22, Val25, and Val29. To obtain detailed information on the structural characteristics of the protein-drug complex, molecular dynamics (MD) simulations were performed using the YASARA software; the analysis of the MD trajectories showed excellent stability of the complex as highlighted in the graphs of Figure S2. It is interesting to note that, at medium HCQ laying, it establishes a new hydrogen bond between the amino nitrogen and the Phe26 residue, with an average distance of 1.82 Å, while the hydroxyl group establishes a hydrogen bond with Leu65, with an average distance of 1.74 Å. Both hydrogen bonds are stable throughout the MD simulations. The re-docking (−10.6 kcal/mol) performed on the average laying confirmed the excellent stability of the complex favored both by the hydrogen bonds and by the hydrophobic interactions inside the central cavity with the Phe26 residue of the pentamer.

CQ adopts a position exactly opposite to that of HCQ (Figure 1). The quinolinic ring is inserted between two *α*-helices of the pentameric structure establishing hydrophobic interactions with Val25, Phe26, Leu27, and Val29, while the small aliphatic chain is oriented within the channel (Figure S1). Among other things, in the tertiary amino group, it does not establish any interaction with the protein residues. During the MD simulation, the CQ moves along the central axis of the ion channel. After 5 ns of MD simulation, the drug stabilizes, establishing both hydrophobic interactions with Phe20, Ala22, Phe23, Val25, Phe26, and a hydrogen bond with Leu19, while the re-docking value (−9.2 kcal/mol) of the average poses confirms the excellent stability of the complex.

The ionic channels undergo significant conformational changes so that they perform their biological role. These membrane proteins have high plasticity, and some amino acids perform essential functions for the passage from closed to open state. During the MD simulation, we noticed a change in the volume of the central tunnel (the CQ led to an increase in the volume of the central lumen of 31.8%), while HCQ reduced the volume of the cavity by 28.14%. Both values refer to the volume of the central cavity of the protein without any ligand inside after 20 ns of MD simulation. We hypothesized that the two drugs interact differently with some central lumen residues, influencing its ability to change its conformation during its functionality. Probably, CQ could destabilize the ion channel structure by removing Phe26 residues from the center of the cavity, causing an expansion of the ion channel; conversely, the hydrophobic interactions of HCQ with the Phe26 (A–E) residues increase the stability of the central pore by bringing the chains of the pentameric structure closer, causing a reduction in the internal volume.

### 2.2. NSP10/NSP14

Nsp14 is a nonstructural protein of coronaviruses. It is known that nsp14 had two different roles showing both a proofreading activity [83] and methylase activity [84]. Nsp14 is a nonstructural CoV-2 protein involved in virus replication fidelity by binding its proofreading subunit to the CoV-2 RNA polymerase [85]. Moreover, it has been shown that the nsp14, through its exonuclease domain, reduced mismatch during replication [86,87,88]. Notably, this mismatch repair activity has been related to the low mutation rate of SARS-CoV [62]. Furthermore, an accurate exonuclease activity represents an essential factor that could affect the activity and use of nucleoside analogs in the treatment of Coronavirus infections [89].

Nsp10, a nonstructural protein of 139 amino acids, is a most conserved protein of the replicative machine of SARS-CoV [90] and has been considered as a crucial multifunctional cofactor in their replication [91]. Although nsp10 is showing no enzyme activity, its role seems to be central to two distinct regulatory mechanisms: In fact, as reported by Bouvet et al., nsp10 interacts with both nsp14 and nsp16 triggering 3′,5′-exoribonuclease and 2′-*O*-methyltransferase activities, respectively [92,93,94,95]. Therefore, an interaction with nsp1, nsp7, nsp13, and itself has been also reported [91,96]. Probably, such interaction with itself may explain why peptides derived from the interaction domain of nsp10 with nsp16 have been shown to inhibit the activity of 2′-*O*-methyltransferase complex nsp10/nsp16 SARS-CoV [96].

The *N*-terminal exoribonuclease (ExoN) domain plays a proofreading role in the prevention of mutagenesis, while the *C*-terminal domain functions as a (guanine-N7) methyltransferase (N7-MTase) for mRNA capping. The nsp10 protein interacts with nsp14 ExoN to stabilize it and stimulate its activity [62]. The cap-precursor guanosine-P3-adenosine-5′,5′-triphosphate, and *S*-adenosyl methionine bind in proximity in a very tight pocket between two *β*-sheets to accomplish methyl transfer. Assembly of a cap1 structure at the 5′ end of viral mRNA assists in translation and evading the host defense [97,98,99]. In the absence of nsp10, nsp14 cannot catalyze nucleotide excision efficiently. The structure of the nsp10/nsp14 complex explains this requirement of nsp10 for the enzymatic activity of nsp14. The extensive interaction of nsp10 with nsp14 suggests that nsp10 might be necessary to maintain the structural stability of the ExoN domain and fully unleash the ExoN activity of nsp14 [62].

We speculated on both domains to find a possible inhibitory activity of CQ and HCQ. Table 1 summarizes the binding energies of the two drugs in both the active sites. HCQ is more active than CQ in both sites, with a binding energy value of −7.0 kcal/mol for the N7-MTase domain and −7.3 kcal/mol for the ExoN domain. The binding energy values for CQ are −6.2 and −6.0 kcal/mol, respectively.

**Figure 3:**
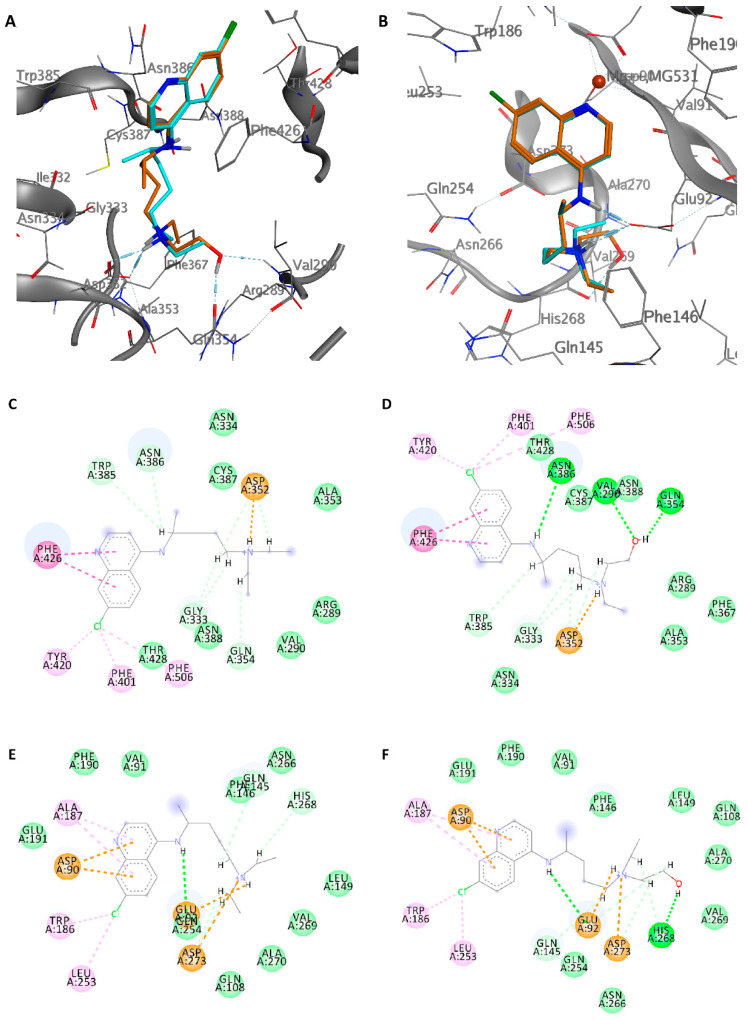
Interaction profile of the best-docked poses for CQ (blue) and HCQ (orange) in the N7-MTase (**A**) and ExoN (**B**) domain. Two-dimensional (2D) interaction diagram of CQ (**C**) and HCQ (**D**) in N7-MTase domain, and CQ (**E**) and HCQ (**F**) in ExoN domain.

As shown in Figure 3, both drugs have an overlapping pose in the N7-MTase domain. The hydrogens of the tertiary amino group form a hydrogen bond with the carboxyl group of Asp352, while the hydroxyl group in the HCQ forms hydrogen bonds with the carbonyl group of Gln354 and the amide hydrogen of Val296 at 1.7 and 2.2 Å, respectively, giving a higher energy binding value. The quinolinic ring, inside the pocket, is stabilized by the *π*-*π* interaction with Phe426, and the chlorine atom establishes hydrophobic interactions with The401, Tyr401, and Phe506. Moreover, for the ExoN domain, the poses of CQ and HCQ are very similar. In both cases, the secondary and tertiary amino nitrogen form a bidentate hydrogen bond with Glu92, while the quinolinic ring establishes cation-*π* interactions with the magnesium atom and Asp90. The hydrophobic and Van der Waals interactions with Val91, Trp186, Ala187, Phe190, Glu191, and Leu253 further stabilize the aromatic region of the ligands.

MD simulations were performed to verify the stability of the complexes. At the N7-MTase site, HCQ showed better stability than CQ throughout the MD simulation period, with less square root mean deviation (RMSD) of both the ligand and the protein system (Figures S3 and S4). The hydrogen bond between the tertiary amine nitrogen atom and Asp352 was stable, with an average length of 1.78 Å, as well as the hydrogen bond between the oxygen of the hydroxyl group and Asn354, with an average length of 1.86 Å. The mean RMSD value of the protein backbone atoms is estimated 1.95 Å for the HCQ complex and 2.14 Å for the CQ one. Although the two systems showed good stability, the interaction with HCQ was more efficient throughout the MD simulation.

The study of the trajectories of the complexes in the ExoN domain allowed us to ascertain better stability of HCQ compared to CQ. The former maintained a better RMDS with an average value of 1.58 Å compared to 1.71 Å of the CQ. Even the RMSD of the protein structure underwent fewer fluctuations than the starting structure. It was interesting to note that HCQ maintained the hydrogen bond bidentate throughout the MD simulation, unlike CQ, which preferred a more linear pose along with the active site (Figures S5 and S6).

**Figure 4:**
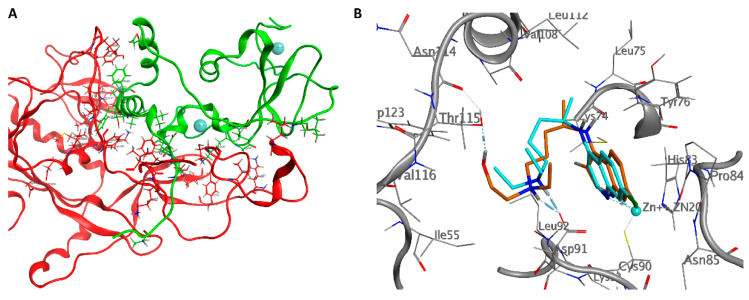
(**A**) Intermolecular interactions between nsp14 (red) is stabilized by nsp10 (green). Zinc ions are represented as blue spheres. (**B**) Docked poses of CQ (blue) and HCQ (orange) are shown with the residues of the binding pocket and the residues interacting with the nsp10 cavity.

In the absence of nsp10, nsp14 cannot catalyze nucleotide excision efficiently [62]. The structure of the nsp10/nsp14 complex explains this requirement of nsp10 for the enzymatic activity of nsp14. The primary residues that contribute to the nsp14-nsp10 interaction belong to two specific regions of the nsp10. The first contact area involves the *N*-terminal ring and the *α*1-helix (Pro1-Leu24) of nsp10, which led to an electron density interpretable for the first nine residues of nsp10. The residues Ala1, Asn3, and Glu6 of nsp10 stabilize the *N*-terminal region of nsp14 forming hydrogen bonds with Lys9, Asp10, and Thr5, while Phe16, Phe19, and Val21 of nsp10 form Van der Waals interactions with Phe60, Met62, and Tyr64 of nsp14. The second area of intermolecular interactions is extensive and includes residues from the ring region after the *α*2-helix and residues close to the zinc atom. Numerous complementary hydrogen bonds are observed here; Asn40, Lys43, Leu45, Thr58, Ser72, Lys93, and Tyr96 of nsp10 interact with Thr25, His26, Cys39, Asp41, Ala23, Tyr51, and His19 of the nsp14 *N*-terminal domain. A salt bridge formed between His80 of nsp10 and Asp126 of nsp14 and a hydrogen bond between Cys90 of nsp10 and Asn129 of nsp14 stabilize the structural elements between *β*5 and *β*6 of nsp14 (Figure 4A). The extensive interactions of the nsp10 with the nsp14 suggest that the nsp10 may be necessary to maintain the structural stability of the ExoN domain of the nsp14 and simultaneously stimulate and maximize its activity [93].

We assumed that the drugs could interact with the nsp10/nsp14 domain by destabilizing the protein-protein complex. The docking results showed no significant interaction in the contact region between the two proteins; however, interesting results were obtained in a small pocket near the zinc atom of nsp10. The two drugs had the following energy binding: −7.7 kcal/mol for HCQ and −6.6 kcal/mol for CQ.

The two drugs have a very similar pose. The nitrogen of the quinolinic ring establishes a coordination bond with the zinc atom, Van der Waals interactions with Cys74, Asn85, and Thr111, and *π*-*π* interactions with Tyr76. The amine forms a salt bridge with Asp91 at 2.06 Å, while the hydroxyl group of HCQ establishes a hydrogen bond with the carbonyl oxygen of Thr115 at 1.97 Å (Figure 4B).

### 2.3. NSP10/NSP16

Nsp16 is a 298 amino acids protein that is involved in methylation of the 2′-hydroxy group of adenines using *S*-adenosylmethionine as a source of methyl [100,101]. However, the nsp16 protein is an RNA cap-modifying enzyme that is devoid of any enzymatic activity, but it is activated by nsp10, which interacts with nsp16 and selectively confers upon its 2′-O-MTase activity on N7-methyl guanine RNA caps [102]. Nsp16 is also involved in the modulation of pathogenesis and type I Interferon susceptibility, as well as it has been suggested as a vaccine candidate [103].

**Figure 5:**
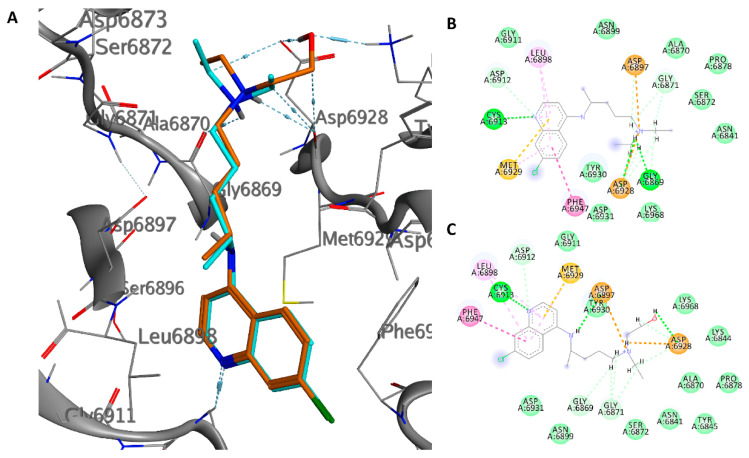
Interaction profile of the best-docked poses for CQ (blue) and HCQ (orange) in the SAM domain of nsp16 (**A**). 2D interaction diagram of CQ (**B**) and HCQ (**C**) in the SAM domain of nsp16.

The crystal structure of the nsp10/nsp16 methyltransferase (PDB ID: 6W6L) of SARS-CoV-2 was used for docking purposes. The docking results we obtained show that the two drugs have a very similar pose inside the pocket that binds SAM, despite the hydroxyquinoline having a better energy binding (−8.1 versus −7.6 kcal/mol). The quinolinic ring shows a hydrogen bond with the amide residue Cys6913 and hydrophobic interactions with Leu6898, Phe6947, and Met6929. The tertiary amino nitrogen atom of CQ forms a salt bridge with Gly9869, while that of HCQ with the carbonyl oxygen of Asp6928; the alcoholic group of HCQ forms hydrogen bonds with the carboxylic residue of Asp6928 (1.87 Å) and the amino group of Lys6869 (1.98 Å) (Figure 5). Probably, the further interaction of the hydroxyl group in the HCQ, inside the enzyme pocket, increases the affinity of the drug.

The average pose in MD simulation showed that HCQ improves interactions with the active site, the hydrogen bonds with Asp6897, and Asp6928 whereas CQ maintains a stable pose demonstrating the low RMSD value (Figures S7 and S8).

## 3. Materials and Methods

### 3.1. Structures Preparation and Minimization

The structures of all the molecules used in this study were built using Marvin Sketch (18.24, ChemAxon Ltd., Budapest, Hungary, http://www.chemaxon.com). A first molecular mechanics energy minimization was used for 3D structures created from the SMLES; the Merck molecular force field (MMFF94) present in Marvin Sketch was used. The protonation states were calculated, assuming a neutral pH. The PM3 Hamiltonian, as implemented in the MOPAC package (MOPAC2016 v. 18.151, Stewart Computational Chemistry, Colorado Springs, CO, USA) [104], was then used to further optimize the 3D structures before the alignment for the docking calculations.

### 3.2. Molecular Docking

Flexible ligand docking experiments were performed by employing the AutoDock 4.2.6 software implemented in YASARA (v. 19.5.5, YASARA Biosciences GmbH, Vienna, Austria) [105,106], using the three-dimensional crystal structure of the SARS coronavirus nsp10/nsp14 complex with functional ligands SAH and GpppA (PDB ID: 5C8S), NMR structure of the SARS coronavirus E protein pentameric ion channel (PDB ID: 5 × 29), and nsp10/nsp16 complex (PDB ID: 6W61) obtained from the Protein Data Bank (PDB, http://www.rcsb.org/pdb), the Lamarckian genetic algorithm (LGA). The crystallized ligand has been eliminated using the YASARA software. The maps were generated by the program AutoGrid (4.2.6) with a spacing of 0.375 Å and dimensions that encompass all atoms extending 5 Å from the surface of the structure of the crystallized ligand. All parameters were inserted at their default settings, as previously reported [107,108]. In the docking tab, the macromolecule and ligand were selected, and GA parameters were set as ga_runs = 100, ga_pop_size = 150, ga_num_evals = 25,000,000, ga_num_generations = 27,000, ga_elitism = 1, ga_mutation_rate = 0.02, ga_crossover_rate = 0.8, ga_crossover_mode = two points, ga_cauchy_alpha = 0.0, ga_cauchy_beta = 1.0, number of generations for picking worst individual = 10.

### 3.3. Molecular Dynamics Simulations

The molecular dynamics simulations of the nsp10/nsp14/ligand complexes and E protein/ligand complexes were performed with the YASARA structure package. A periodic simulation cell with boundaries extending 8 Å [109] from the surface of the complex was employed. The box was filled with water, with a maximum sum of all water bumps of 1.0 Å, and a density of 0.997 g mL^−1^.

The setup included an optimization of the hydrogen bonding network [110] to increase the solute stability, and a p*K*_a_ prediction to fine-tune the protonation states of protein residues at the chosen pH of 7.4 [111]. NaCl ions were added with a physiological concentration of 0.9%, with an excess of either Na or Cl to neutralize the cell. Water molecules were deleted to readjust the solvent density to 0.997 g/mL. The final system dimensions were approximately 80 × 80 × 130 Å^3^ for nsp10/nsp14/ligand complexes and 94 × 78 × 94 Å^3^ for E protein/ligand complexes.

To best represent the biological environment, for E protein/ligand complexes, each of the best pose ligand/receptor complex structure was immersed in a simulated bilayer membrane, in the above reported physiological environment conditions, and subjected to a molecular dynamics (MD) simulation. The simulation was set up automatically by first scanning the protein for exposed transmembrane helices (i.e., helices longer than 16 residues, with more than seven hydrophobic residues and more than three exposed ones (accessible side-chain surface area >30% of maximum)). The major axis vectors of these helices (i.e., the direction vectors of the least-squares lines through the C_alpha_ atoms) were summed up to obtain the major axis of the protein, which was then oriented along the *Y*-axis, generally with respect to the plane of the membrane and the XZ plane. The best shift of the membrane along this major axis was obtained by scanning the protein for the region with the largest number of exposed hydrophobic residues (see definition above) and a width of 28 Å (corresponding to the membrane core).

Having placed an equilibrated membrane structure (consisting of 50% of phosphatidylcholine and 50% of phosphatidylethanolamine) at this location named ‘MemCenterY’, the system was enclosed in a simulation cell of size (100 × 80 × 100) Å, and the protein was temporarily scaled by 0.9 along the X–Z axes, and then, strongly clashing membrane lipids were deleted (lipids with an atom closer than 0.75 Å to a protein atom). The temporary protein scaling, which was needed to avoid the deletion of too many lipids around the protein, was then slowly removed during a short simulation at 298 K in vacuum. The protein (with all of the atoms kept fixed) was scaled by 1.02 along the X–Z axes every 200 fs, while the membrane was allowed to move, but was restrained to ideal geometry (by pulling lipid residues with an atom further than 21.5 Å away from MemCenterY back into the membrane, and by pushing phosphorus atoms closer than 14 Å to MemCenterY back outwards).

The simulations were run using the ff14SB force field [112] for the solute, with Lipid17, GAFF2 [113], and AM1BCC [114] for non-standard residues, and TIP3P for water.

As soon as the protein had reached its original size again, the protein side-chain p*K*_a_s were predicted, protonation states were assigned according to pH 7.4, and the simulation cell was filled with water, 0.9% NaCl, and counter ions.

The main simulation was then run with a cutoff of 8 Å for Van der Waals forces (the default used by AMBER) [115], and no cutoff was applied to electrostatic forces (using the Particle Mesh Ewald algorithm) [116], a four fs time-step, constrained hydrogen atoms, and at constant pressure and temperature (NPT ensemble) using algorithms described in detail previously [117]. The ligand force field parameters were generated with the AutoSMILES utility [114], which employs semiempirical AM1 geometry optimization and assignment of charges, followed by assignment of the AM1BCC atom and bond types with refinement using the RESP charges, and finally the assignments of general AMBER force field atom types. Optimization of the hydrogen bond network of the various enzyme-ligand complexes was obtained using the method established by Hooft et al. [110], in order to address ambiguities arising from multiple side-chain conformations and protonation states that are not well resolved in the electron density [118]. During the initial 250 ps, the membrane was restrained to avoid distortions while the simulation cell adapted to the pressure exerted by the membrane (see above; additionally, water molecules that got closer than 14 Å to MemCenterY were pushed outside). The source code of this simulation protocol and visualizations of the individual steps can be found at www.yasara.org/membranemd.

After the membrane placement, a short MD was run on the solvent only. The entire system was then energy minimized using first a steepest descent minimization to remove conformational stress, followed by a simulated annealing minimization until convergence (<0.01 kcal/mol Å). The MD simulation was then initiated, using the NPT ensemble at 298 K, and integration time steps for intramolecular and intermolecular forces every 1.25 and 2.5 fs, respectively.

Finally, 20 ns MD simulations without any restrictions were conducted, and the conformations of each system were recorded every 200 ps. On the averaged structure of the last 3 ns frames, a second cycle of energy minimization, identical to the first, was applied. After inspection of the solute RMSD as a function of simulation time, the last 3 ns averaged structures were considered for further analysis.

## 4. Conclusions

The antiviral effect of HCQ against the SARS-CoV-2 infection compared to QC in vitro has been demonstrated [119], although there are still controversial opinions on the clinical effect of these drugs. Our results suggested that HCQ has a better energy binding with the examined targets than CQ, mainly due to the hydrogen bonding of the hydroxyl group in HCQ with polar amino acid residues. E protein functionality, necessary in virus maturation processes, could be disrupted by CQ and HCQ by interaction in the central cavity of the ion channel. Although the docking poses have a different orientation within the central lumen showing interactions with different regions of the protein, these can influence the flexibility of the protein structure. Furthermore, the results obtained hinted that proofreading and capping of viral RNA in SARS-CoV-2, carried out by nsp10/nsp14 and nsp10/nsp16, could be influenced by CQ and HCQ. The energy binding for nsp16 in the SAM domain was slightly higher than the viral regions of nsp14. MD simulation studies demonstrate the stability of drug interactions with the protein regions analyzed. Although computational studies deserve more attention for nsp10, we have shown that CQ and HQC have a good affinity with an area of the viral protein that could influence the cofactor effect against nsp14 and nsp16.

## Figures and Tables

**Table 1:** Calculated free binding energies (Δ*G*_B_, in kcal/mol) of CQ and HCQ in N7-MTase and ExoN domains.

Compound	N7-MTase (kcal/mol)	ExoN (kcal/mol)
CQ	−6.2	−6.0
HCQ	−7.0	−7.3

## Supplementary Materials

Supplementary materials can be found at https://www.mdpi.com/1422-0067/21/16/5856/s1.
