# Supplementary material for: New Anti SARS-Cov-2 Targets for Quinoline Derivatives Chloroquine and Hydroxychloroquine
Source: Int J Mol Sci. 2020 Aug 14;21(16):5856. doi: 10.3390/ijms21165856 (PMC7461590; doi:10.3390/ijms21165856)
Supplement: Supplementary file 1 [file ijms-21-05856-s001.pdf]

International Journal of *Molecular Sciences*

MDPI

Supplementary Material

New Anti SARS-Cov-2 Targets for Quinoline Derivatives Chloroquine and Hydroxychloroquine

Davide Gentile [1], Virginia Fuochi [2], Antonio Rescifina [1,*] and Pio Maria Furneri [2,*]

[1] Dipartimento di Scienze del Farmaco, University of Catania, Catania, Italy; davide.gentile@unict.it (D.G.) arescifina@unict.it (A.R.)
[2] Dipartimento di Scienze Biomediche e Biotecnologiche, University of Catania, Catania, Italy; vfuochi@unict.it (V.F.) furneri@unict.it (P.M.F.)
* Correspondence: arescifina@unict.it (A.R.); furneri@unict.it (P.M.F.)

Table of contents

**Figure S1.** E Protein-CQ. Total energy (up-left) and RMSDs (up-right) of protein and its complexes with the ligand. Docking binding pose (down-left) and interaction (down-right) inside the binding pocket of the protein.	S2
**Figure S2.** E Protein-HCQ. Total energy (up-left) and RMSDs (up-right) of protein and its complexes with the ligand. Docking binding pose (down-left) and interaction (down-right) inside the binding pocket of the protein.	S2
**Figure S3.** NSP14-CQ N7 MTase domain. Total energy (up-left) and RMSDs (up-right) of enzyme and its complexes with the ligand. Docking binding pose (down-left) and interaction (down-right) inside the binding pocket of the enzyme.	S3
**Figure S4.** NSP14-HCQ N7-MTase domain. Total energy (up-left) and RMSDs (up-right) of enzyme and its complexes with the ligand. Docking binding pose (down-left) and interaction (down-right) inside the binding pocket of the enzyme.	S3
**Figure S5.** NSP14-CQ ExoN domain. Total energy (up-left) and RMSDs (up-right) of enzyme and its complexes with the ligand. Docking binding pose (down-left) and interaction (down-right) inside the binding pocket of the enzyme.	S4
**Figure S6.** NSP14-HCQ ExoN domain. Total energy (up-left) and RMSDs (up-right) of enzyme and its complexes with the ligand. Docking binding pose (down-left) and interaction (down-right) inside the binding pocket of the enzyme.	S4
**Figure S7.** NSP16-CQ SAM domain. Total energy (up-left) and RMSDs (up-right) of enzyme and its complexes with the ligand. Docking binding pose (down-left) and interaction (down-right) inside the binding pocket of the enzyme.	S5
**Figure S8.** NSP16-HCQ SAM domain. Total energy (up-left) and RMSDs (up-right) of enzyme and its complexes with the ligand. Docking binding pose (down-left) and interaction (down-right) inside the binding pocket of the enzyme.	S5

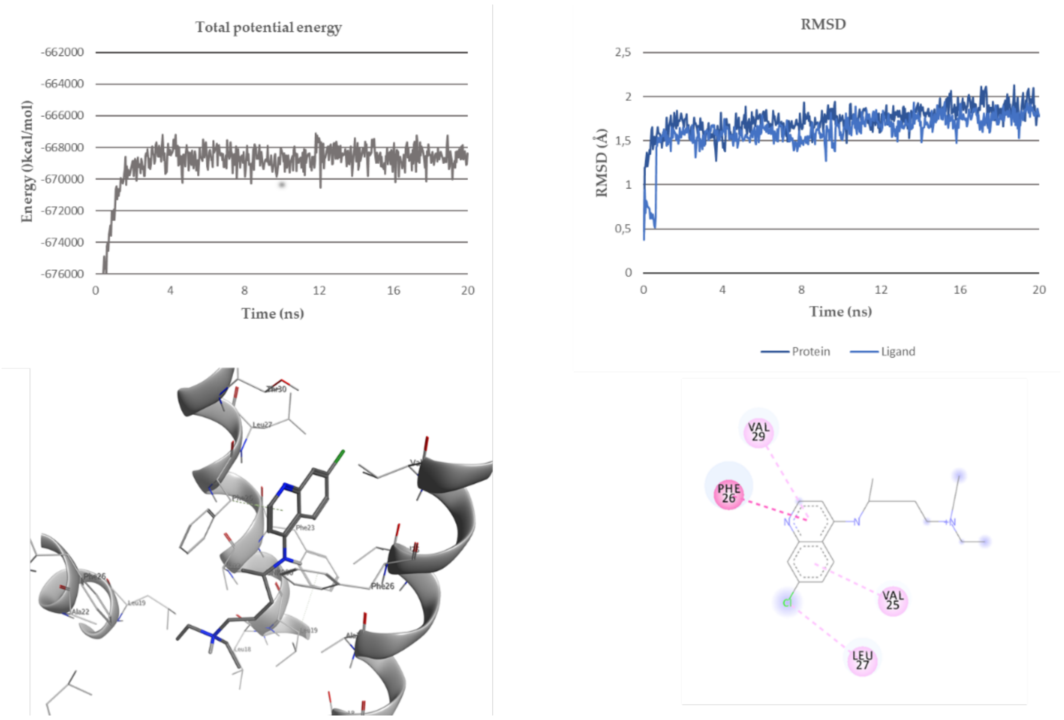

Figure S1. E Protein-CQ. Total energy (up-left) and RMSDs (up-right) of protein and its complexes with the ligand. Docking binding pose (down-left) and interaction (down-right) inside the binding pocket of the protein.

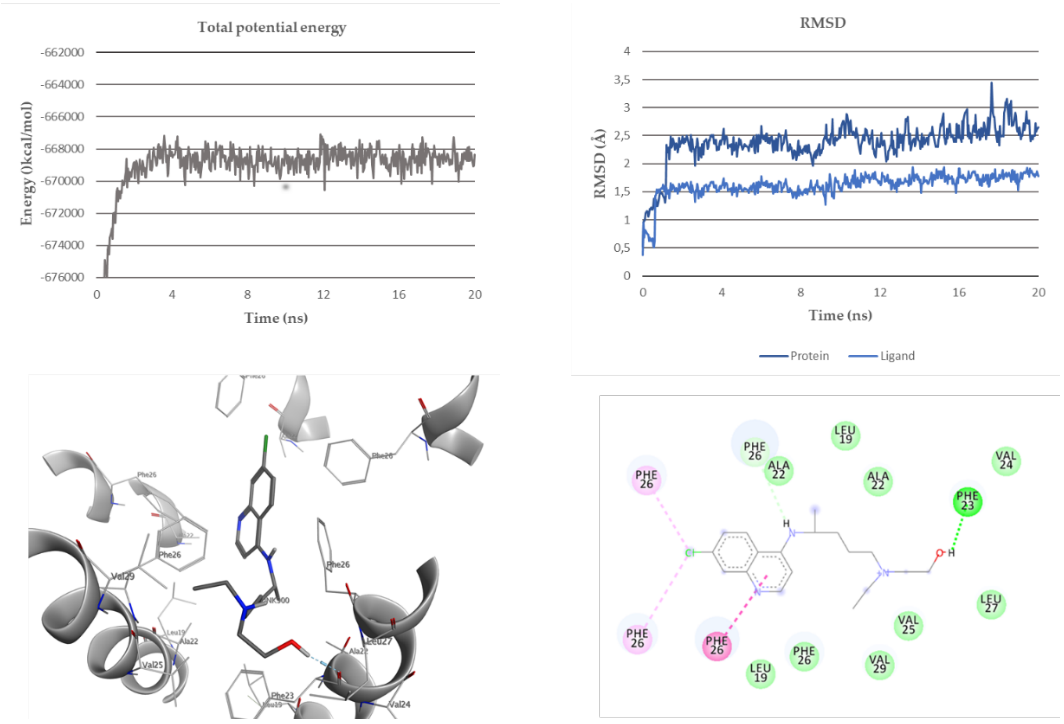

Figure S2. E Protein-HCQ. Total energy (up-left) and RMSDs (up-right) of protein and its complexes with the ligand. Docking binding pose (down-left) and interaction (down-right) inside the binding pocket of the protein.

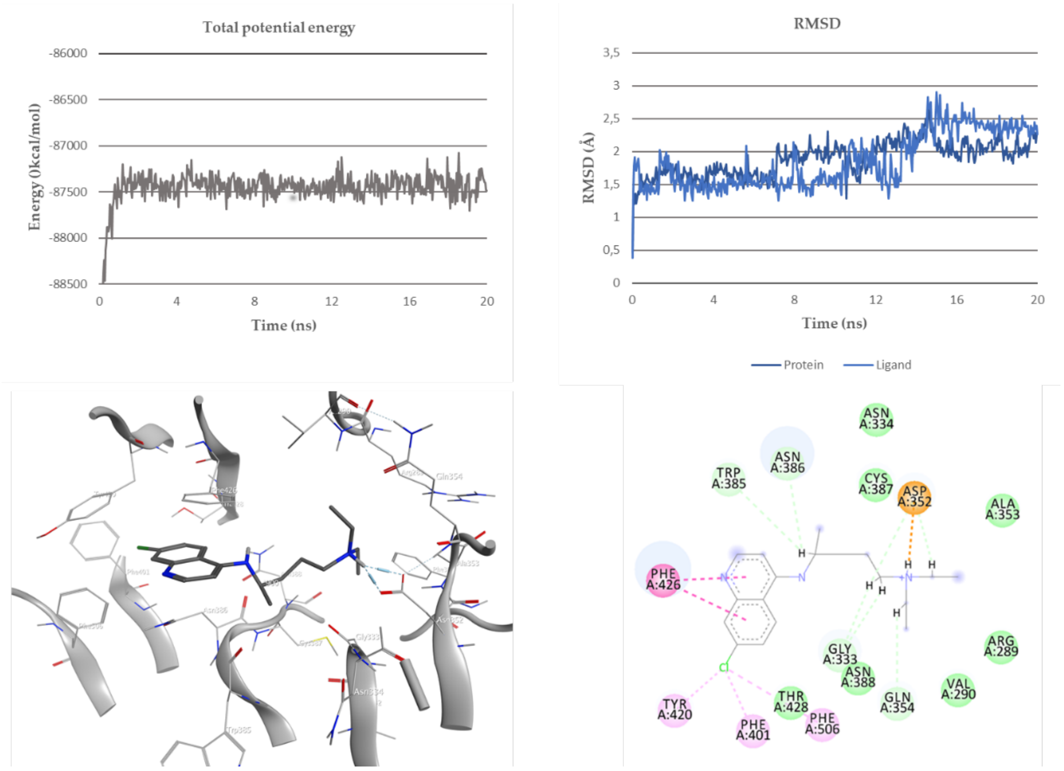

Figure S3. NSP14-CQ N7 MTase domain. Total energy (up-left) and RMSDs (up-right) of enzyme and its complexes with the ligand. Docking binding pose (down-left) and interaction (down-right) inside the binding pocket of the enzyme.

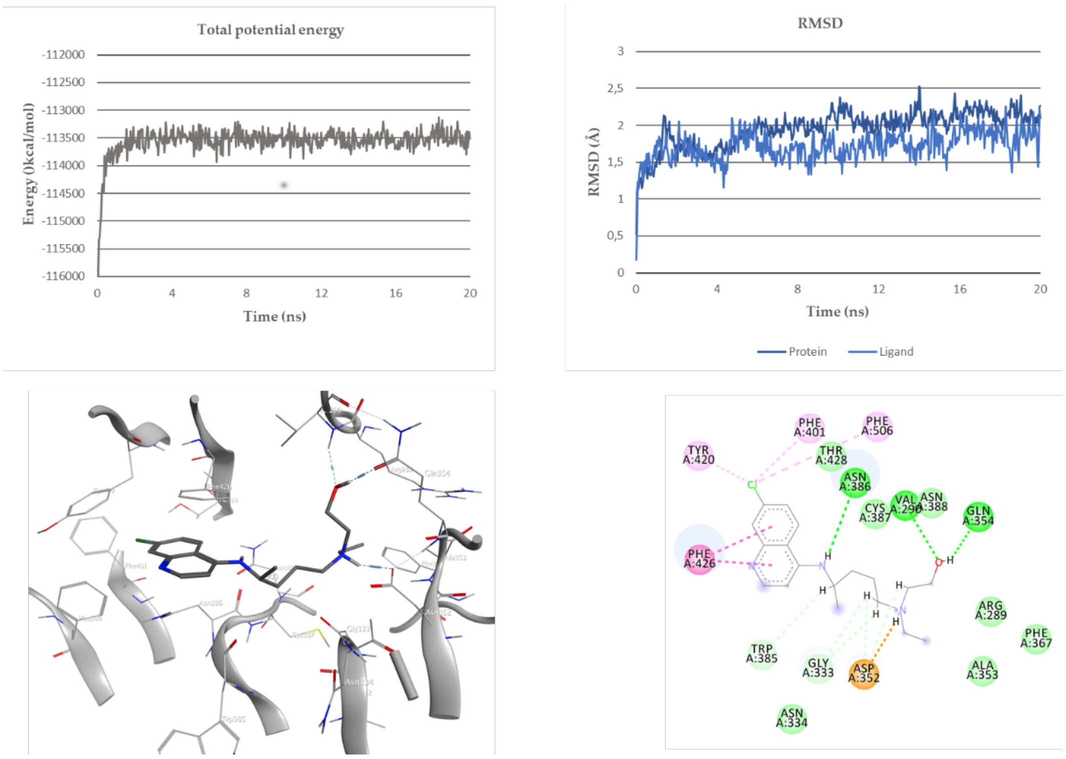

Figure S4. NSP14-HCQ N7-MTase domain. Total energy (up-left) and RMSDs (up-right) of enzyme and its complexes with the ligand. Docking binding pose (down-left) and interaction (down-right) inside the binding pocket of the enzyme.

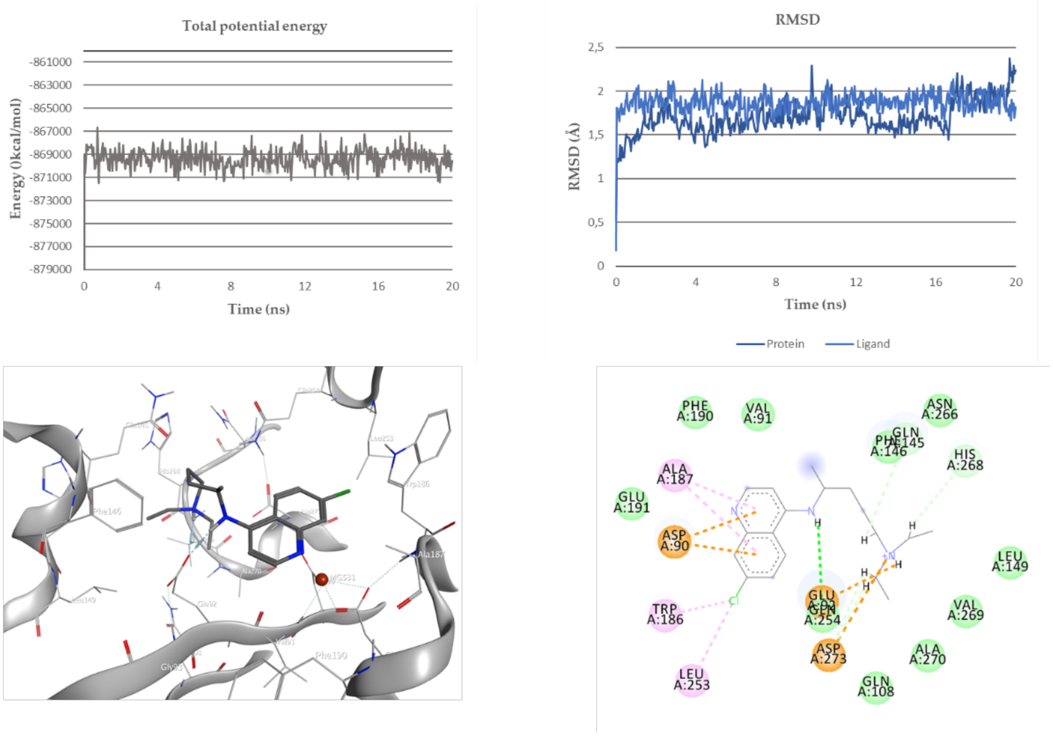

Figure S5. NSP14-CQ ExoN domain. Total energy (up-left) and RMSDs (up-right) of enzyme and its complexes with the ligand. Docking binding pose (down-left) and interaction (down-right) inside the binding pocket of the enzyme.

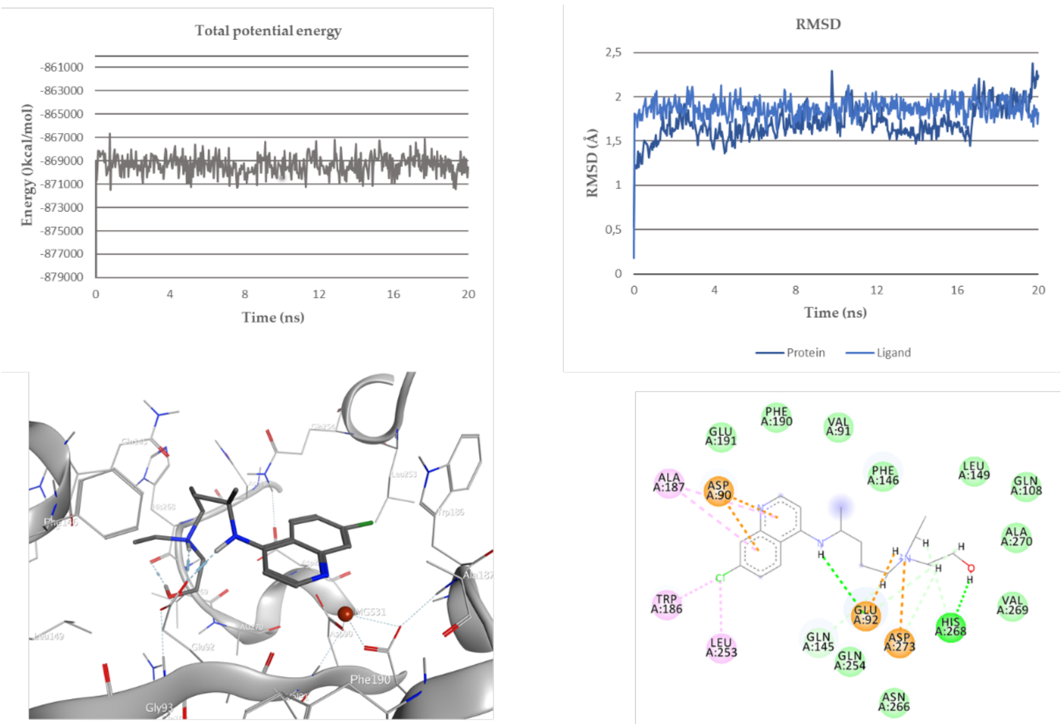

Figure S6. NSP14-HCQ ExoN domain. Total energy (up-left) and RMSDs (up-right) of enzyme and its complexes with the ligand. Docking binding pose (down-left) and interaction (down-right) inside the binding pocket of the enzyme.

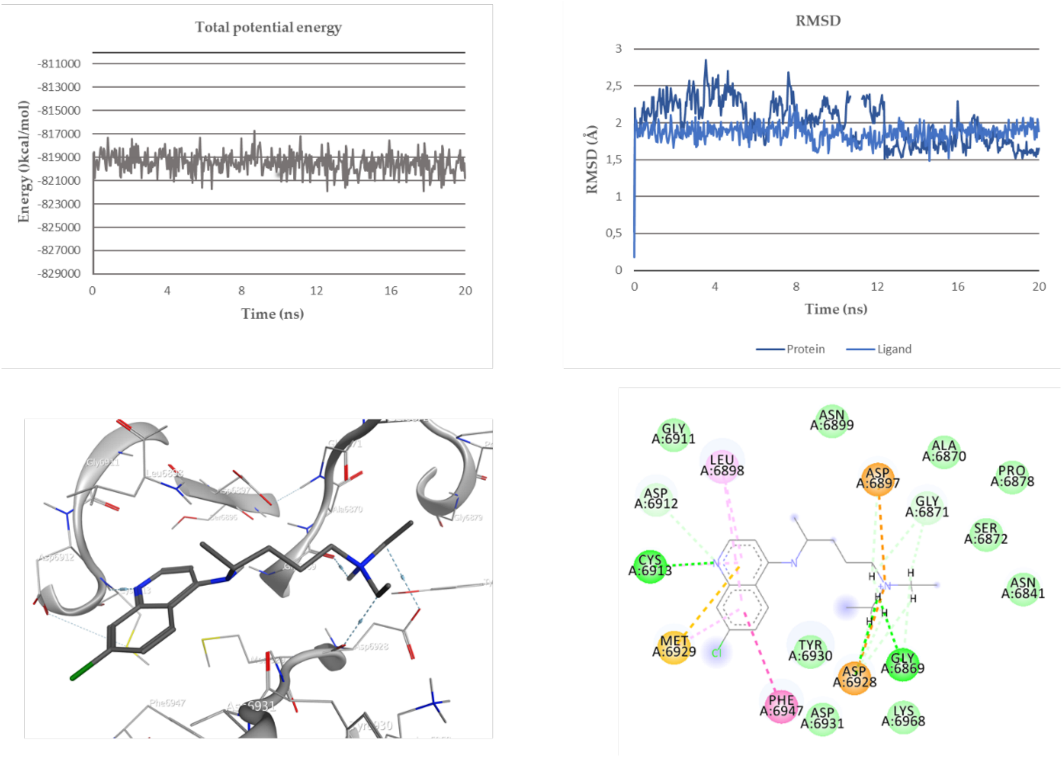

Figure S7. NSP16-CQ SAM domain. Total energy (up-left) and RMSDs (up-right) of enzyme and its complexes with the ligand. Docking binding pose (down-left) and interaction (down-right) inside the binding pocket of the enzyme.

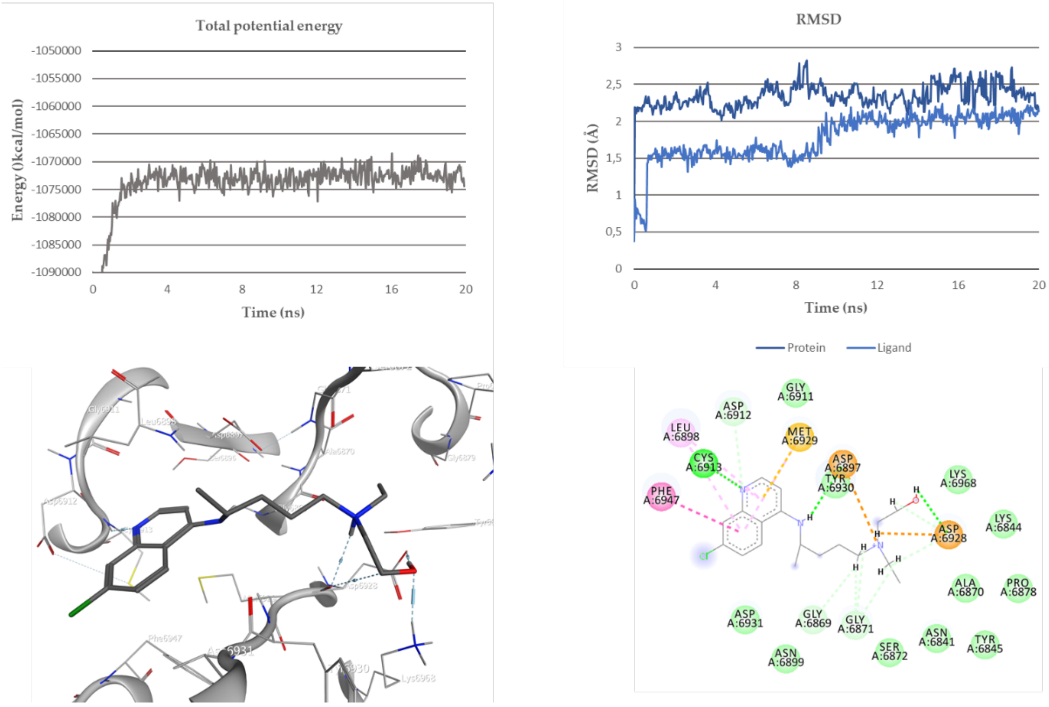

Figure S8. NSP16-HCQ SAM domain. Total energy (up-left) and RMSDs (up-right) of enzyme and its complexes with the ligand. Docking binding pose (down-left) and interaction (down-right) inside the binding pocket of the enzyme.
